# Female Meiotic Sex Chromosome Inactivation in Chicken

**DOI:** 10.1371/journal.pgen.1000466

**Published:** 2009-05-22

**Authors:** Sam Schoenmakers, Evelyne Wassenaar, Jos W. Hoogerbrugge, Joop S. E. Laven, J. Anton Grootegoed, Willy M. Baarends

**Affiliations:** 1 Department of Reproduction and Development, Erasmus MC, University Medical Center, Rotterdam, The Netherlands; 2 Department of Obstetrics and Gynaecology, Erasmus MC, University Medical Center, Rotterdam, The Netherlands; Massachusetts General Hospital, Howard Hughes Medical Institute, United States of America

## Abstract

During meiotic prophase in male mammals, the heterologous X and Y chromosomes remain largely unsynapsed, and meiotic sex chromosome inactivation (MSCI) leads to formation of the transcriptionally silenced XY body. In birds, the heterogametic sex is female, carrying Z and W chromosomes (ZW), whereas males have the homogametic ZZ constitution. During chicken oogenesis, the heterologous ZW pair reaches a state of complete heterologous synapsis, and this might enable maintenance of transcription of Z- and W chromosomal genes during meiotic prophase. Herein, we show that the ZW pair is transiently silenced, from early pachytene to early diplotene using immunocytochemistry and gene expression analyses. We propose that ZW inactivation is most likely achieved via spreading of heterochromatin from the W on the Z chromosome. Also, persistent meiotic DNA double-strand breaks (DSBs) may contribute to silencing of Z. Surprisingly, γH2AX, a marker of DSBs, and also the earliest histone modification that is associated with XY body formation in mammalian and marsupial spermatocytes, does not cover the ZW during the synapsed stage. However, when the ZW pair starts to desynapse, a second wave of γH2AX accumulates on the unsynapsed regions of Z, which also show a reappearance of the DSB repair protein RAD51. This indicates that repair of meiotic DSBs on the heterologous part of Z is postponed until late pachytene/diplotene, possibly to avoid recombination with regions on the heterologously synapsed W chromosome. Two days after entering diplotene, the Z looses γH2AX and shows reactivation. This is the first report of meiotic sex chromosome inactivation in a species with female heterogamety, providing evidence that this mechanism is not specific to spermatogenesis. It also indicates the presence of an evolutionary force that drives meiotic sex chromosome inactivation independent of the final achievement of synapsis.

## Author Summary

Meiosis is a sequence of two specialized cell divisions during which haploid gametes are generated. During meiotic prophase, homologous chromosomes pair and recombine to allow proper separation of chromosomes during the first meiotic division. The pairing mechanism is challenged by the presence of the largely nonhomologous sex chromosomes in spermatocytes of male mammals, since X and Y pair only in the short regions of homology. The unpaired nonhomologous regions are recognized and transcriptionally silenced, which leads to the formation of the so-called XY body. In mammalian females, which carry two homologous X chromosomes, no such structure is formed and the sex chromosomes are both active in oocytes. We asked whether meiotic silencing of sex chromosomes also occurs during gametogenesis in chickens. In this species, males carry two Z chromosomes, and females are ZW. We show that Z and W fully pair in oocytes, despite the overall lack of sequence homology. Surprisingly, the ZW pair is transcriptionally silenced during meiotic prophase and remains inactive until the two chromosomes have largely separated. Reactivation of Z at this stage may be necessary to allow expression of genes that are required for further oocyte development. These data show that meiotic sex chromosome silencing occurs also in species with female heterogamety.

## Introduction

During meiotic prophase, homologous chromosomes pair and are held together by the synaptonemal complex (reviewed in [1]). In spermatocytes of male mammals, the heterologous X and Y chromosomes pair and synapse only in small pseudoautosomal regions (PARs). The presence of the largely unsynapsed X and Y chromosomal axes is associated with meiotic sex chromosome inactivation (MSCI) [2],[3]. The two X chromosomes in meiotic prophase in oocytes show complete synapsis and are transcriptionally active.

In birds, females are heterogametic, carrying Z and W chromosomes (ZW), whereas males have the homogametic ZZ constitution. The chicken Z chromosome is the larger of the two chromosomes (http://www.ensembl.org/Gallus_gallus/index.html). Similar to the mammalian X and Y sex chromosomes, the Z and W chromosomes share only a small pseudoautosomal region [4]. However, the behaviour of the ZW pair during female oogenesis in the chicken differs from that of the XY pair in mammalian spermatocytes, in that the ZW chromosomes appear to reach a stage of complete synapsis. Based on electron micrographs, Solari [5] analysed the pairing between Z and W throughout the pachytene stage and found that the chromosomal axes of the Z chromosome thickens and shortens (most likely by folding back on itself), and wraps itself around the W chromosome to achieve complete synapsis during the brief so-called equalized stage. Subsequently, the Z and W chromosomes desynapse but remain attached at their tips when the oocytes enter diplotene. The morphological changes of the Z and W axes have been explained by a mechanism called synaptic adjustment [5]. This mechanism describes the process of resolving an axial length difference between aligned chromosomes to achieve complete synapsis [6],[7].

During mitotic prophase in female chicken cells, the small W chromosome appears to be heterochromatic [8] indicating that the W chromosome is mostly inactive in somatic cells. During early meiotic prophase in leptotene and zygotene oocytes, such a heteropycnotic area appears to be absent [9]–[11]. Subsequently, Z and W pair completely. Although the pairing is mainly heterologous, Jablonka and Lamb [12] have suggested that pairing, synapsis and subsequent retention of an active state is preferred above meiotic inactivation of Z and W, because of a requirement for Z- and/or W-linked genes for maintenance and growth of the large and long-living oocytes. However, Solari [10] describes the appearance of a more dense chromatin structure surrounding the ZW pair in late pachytene and early diplotene oocytes, and the appearance of a heteropycnotic body in some late pachytene and diplotene nuclei of chicken oocytes. This observation suggests that some form of Z and/or W inactivation may occur during late meiotic prophase.

MSCI in mammals is thought to be a specialization of a more general process that silences unsynapsed chromatin during meiotic prophase, named MSUC (meiotic silencing of unsynapsed chromatin) [13]–[15]. Similar, but mechanistically distinct mechanisms (meiotic silencing by unpaired DNA; MSUD) are operative in a variety of distant species such as *Caenorhabditis elegans* and *Neurospora crassa* (reviewed in [16]).

In mammalian meiosis, chromosomal alignment and pairing is preceded by induction of DNA double strand breaks (DSBs) by the topoisomerase-like protein SPO11, and these DSBs are thought to participate in homology recognition [17],[18]. After formation of DSBs, the homologous recombination repair protein RAD51 rapidly forms filaments on the 3′ end single-strand DNA overhangs of meiotic DSBs [19]. The presence of persistent RAD51 foci on the unpaired X chromosome of mouse and man indicates that DSBs in heterologous regions show delayed repair [19]–[21]. This is most likely due to the fact that a non-sister chromatid from a homologous chromosome is not available for strand invasion and recombination repair. Ashley et al. [20] reported a high concentration of RAD51 foci on the unsynapsed axis of the Z chromosome in chicken oocytes during early pachytene, which disappear as the oocytes progress through pachytene. Unsynapsed sex chromatin, persistent DSBs, and meiotic silencing are always associated in mice [13],[15],[22]. In chicken oocytes, however, the ZW pair reaches a state of complete synapsis, but possibly with persistent DSBs. In the present paper, we have investigated whether meiotic DSBs in chicken oocytes persist on the Z chromosome, analogous to persistence of X-chromosomal meiotic DSBs in mouse spermatocytes, and whether or not this would be associated with MSCI.

## Materials and Methods

### Isolation of Oocytes from Chicken Ovaries

Oocytes were isolated from embryonic day 20 (E20), day 4 (P4) and 7 (P7) post hatching female chickens. Ovaries were collected and incubated for 30 min in 20 ml Dulbecco's-PBS medium containing 1 mg/ml collagenase, 1 mg/ml trypsin and 0,5 mg/ml hyaluronidase (Worthington, Lakewood, USA) in a shaking waterbath with an amplitude of 1 cm at 37°C (60 cpm/min). A single cell suspension was obtained by repeated pipetting of the suspension. After filtration through 70 µm gauze, the cell suspension was centrifuged for 3 min at 800 g. 1 ml of cell suspension in DMEMF12 was loaded on 9 ml of a 3-step gradient of 1.012, 1.037 and 1.071 mg/ml Nycodenz (Nycoprep™ Universal, Axis Shield PoC AS, Oslo, Norway) and centrifuged at 2400 g for 20 min at 20°C. The oocyte fraction was collected from the 1,037 mg/ml layer, centrifuged for 3 min at 800 g and the pellet was snap-frozen in liquid nitrogen and stored at −80°C. Based on SYCP3 staining of spread nuclei preparations from the purified fractions we calculated a purity of 70%, 40% and 50% oocytes in fractions isolated from E20, P4 and P7, respectively.

### Spreads and Immunocytochemistry

Chicken (*Gallus gallus domesticus*) eggs were incubated at 37°C and a humidity of 70–80% until hatching. Chickens were killed by CO_2_ intoxication. The functional left ovary or left and right testes were dissected and placed in Hanks' solution. Spread nuclei preparations of chicken oocytes and spermatocytes were prepared using a modification of the drying-down technique described by Peters et al. [23]. Briefly, ovaries and testes were minced in pieces with forceps and cells were suspended in 500 µl of 100 mM sucrose, containing EDTA-free complete protease inhibitor cocktail (Roche Diagnostics, Almere, The Netherlands). Oocytes and spermatocytes were dispersed on a glass slide dipped in 1% paraformaldehyde fixative with 0.1% Triton X100. After two hours in a humid chamber at room temperature, the slides were allowed to dry for 30 minutes at room temperature, followed by a single wash in 0.08% Photoflo (Kodak, Paris, France) and air-dried. The slides were stored at −80°C.

For immunocytochemistry, frozen slides were defrosted at room temperature and washed with PBS. The slides were blocked with PBS containing 0.5% w/v BSA and 0.5% w/v milk powder, and double stained with different combinations of the following antibodies: rabbit polyclonal anti-SYCP3 (1∶1000), rabbit polyclonal anti-SYCP1 (1∶200) (gifts from C. Heyting, Wageningen), mouse polyclonal anti-γH2AX (1∶1000) (Upstate, Walthum, MA, USA), rabbit polyclonal anti-γH2AX (1∶1000) (Upstate), mouse monoclonal IgM anti-H2AK119ub1 (1∶1000) (Upstate), mouse monoclonal anti-RNA polymerase II, (8WG16) directed against the RNA polymerase II CTD repeat YSPTSPS (1∶600) (Abcam, Cambridge, United Kingdom), mouse monoclonal anti-H4K16ac (1∶200) (Upstate), mouse monoclonal anti-H3K27me3 (1∶100) (Abcam), rabbit polyclonal anti-H3K9me3 (1∶500) (Upstate), and rabbit anti-human RAD51 (1∶500) [24]. For mouse monoclonal primary antibodies, the secondary antibodies were fluorescein isothiocyanate (FITC)-labeled goat anti-mouse IgG antibodies (1∶128) (Sigma, St Louis, USA) for anti-RNA polymerase II, anti-γH2AX, and anti-H3K27me3, FITC-labeled goat anti-mouse IgM (1∶128) (Sigma) for anti-H2AK119ub1 and tetramethylrhodamine isothiocyanate (TRITC)-labeled goat anti-mouse IgG antibodies (1∶128) (Sigma) for anti-γH2AX. The secondary antibody for polyclonal rabbit primary antibodies was tetramethylrhodamine isothiocyanate (TRITC)-labeled goat anti-rabbit IgG antibodies (1∶200) (Sigma) for anti-SYCP3 and fluorescein isothiocyanate (FITC)-labeled goat anti-rabbit IgG antibodies (1∶80) (Sigma) for anti-Rad51, anti-SYCP1, and anti-γH2AX. Primary antibodies were diluted in 10% w/v BSA in PBS and incubated overnight in a humid chamber. Thereafter, slides were washed in PBS, blocked in 10% v/v normal goat serum (Sigma) in blocking buffer (5% milk powder (w/v) in PBS, centrifuged at 13.200 rpm for 10 min), and incubated with secondary antibodies in 10% v/v normal goat serum in blocking buffer at room temperature for 2 hours. Next, the slides were washed in PBS and embedded in Vectashield containing DAPI (4′,6′-diamindino-2-phenylindole) (Vector Laboratories, Burlingame CA, USA). Double stainings of SYCP1 with SYCP3, of RAD51 with SYCP3, and of SYCP3 with H3K9me3 (all rabbit polyclonal antibodies) were obtained by sequential immunostainings with the single antibodies. Images of SYCP1, RAD51 and SYCP3 stainings respectively, were obtained prior to immunostaining with anti-SYCP3 or H3K9me3 of the same nuclei.

### Real-Time RT-PCR

For real-time RT-PCR, RNA was prepared from embryonic female liver, embryonic day (E20), post hatching day 4 (P4) and day 7 (P7) ovaries and oocyte fractions by Trizol (Invitrogen, Breda, The Netherlands), DNase-treated and reverse transcribed using random hexamer primers and Superscript II reverse transcriptase (Invitrogen). PCR was carried out with the Fast SYBR green PCR mastermix (Applied Biosystems, Foster City, USA) in the DNA engine Opticon 2 real-time PCR detection system (Bio-Rad, Hercules, USA). For *ACTB*, *SYCP3, SPO11,* W genes: *NIBPL, SPIN, SMAD2, HINTW* and Z genes*: NIBPL, SPIN1, SMAD2, HINT1, DMRT1, TXNL1, TXN, ILR7, PARP8, SLCA1A3* we used the following conditions: 3 minutes 95°C, then 10 seconds 95°C, 30 seconds 58°C, 30 seconds 72°C for 40 cycles, experiments were performed in triplicate. For data analysis, the average threshold cycle (*C*t) was converted to absolute amount of transcript (*E^−C^* ^t^) (E = efficiency determined via a standard curve) and presented as *E^ C^* ^t Actin -*C*t gene of interest^. To estimate the expression of Z and W encoded genes in oocytes and to correct for differences in purity, we used the following formulas:
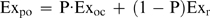


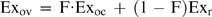
Ex_po_ = measured expression level in the purified oocyte sample, P = purity of the oocytes (0.7, 0.4 and 0.5 for E20, P4 and P7 respectively), Ex_oc_ = expression level in oocytes, Ex_r_ = expression level in the rest of the ovarian cells, Ex_ov_ = measured expression level in the ovary, F = oocyte fraction in the ovary. We equalized the Ex_r_ for *SYCP3* to the expression measured in embryonic liver. This allowed us to calculate the value of F in the different ovary samples. The median value of F was found to be 0.06, and this number was used to calculate Ex_oc_. All –RT reactions were negative. Forward and reverse primers (5′ to 3′): See Table 1.

**Table 1:** Primers used for real-time RT-PCR analyses.

Gene	Forward primer (5′–3′)	Reverse primer (5′-3′)
autosomal		
*SYCP3*	AGAGCATGGAAGAGCTAGAG	AGAGCATGGAAGAGCTAGAG
*SPO11*	AGAAGTGACTGCCCTGCAAC	TGGCTACCAAACAGGAGCTT
W chromosomal genes		
*NIBPL*	AAAGTCCTGCGGGATATGTG	ATGGGACTGGACACTGAAGC
*SPIN*	TCAGCCACGAAGAAACATTG	TGTCCCTTCCATTGTGTTA
*SMAD2*	ACCAGAAACACCACCTCCAG	TTGGTTCAACTGCTGGTCAC
*HINTW*	CTTCTTGGGCGTTTGATGAT	GCGGTAGTCTGAAGGGACTG
Z chromosomal genes		
*NIBPL*	CAGGGTCTCATCCATCCTGT	TCGCATAGAAGGCTCTGGAT
*SPIN1*	GTCTCTGCCAGCATGATGAA	CACTCCCTTCTTTCCATCCA
*SMAD2*	GTCTCTGCCAGCATGATGAA	GTCCCCAAATTTCAGAGCAA
*HINT1*	GTTTTGAATGAAGGGCCTGA	CATGCAGCATCTCTTGTGGT
*DMRT1*	AGTGGCAGATGAAAGGGATG	CGAGGCCAGTATCTGTGTGA
*TXNL1*	GCCCTGGAACTAACACCAGA	TCCCCGTGATTAGACTGGAC
*TXN*	AGAACGGAAAGAAGGTGCAG	AGACATGCTCCGATGTCTCC
*IL7R*	TTCCTACAGCAGCCTGACCT	TGGTACACACAGCCAGGGTA
*PARP8*	CACCAGCCAAAGAATCCAAT	CAGGATGGAATGCCAGTTTT
*SLC1A3*	TCTTGGATCGTCTCCGTACC	CTTCAGCTCATGCCGTGATA

### Fluorescent In Situ Hybridisation (FISH)

First, immunocytochemistry was performed as described above, and images were made of selected nuclei. Probe mixture of digoxigenin-labelled GGA (Gallus GAllus) W and biotin-labelled GGA Z chromosome (heterochromatic part) painting probes (Farmachrom, Kent, UK), salmon sperm DNA and hybridisation buffer were mixed and denatured at 75°C. Slides were treated with 0.005% pepsine solution for 5 minutes at 37°C, washed in 2×SSC at room temperature for 5 minutes, rinsed in distilled water and then air dried. Next, they were dehydrated, air-dried and incubated for 1 hour at 75°C. Again, slides were dehydrated and air dried. Subsequently, RNAse mix (100 µg/ml in PBS) was placed on each slide, and slides were incubated in a humid chamber at 37°C. After 1 hour, slides were again air dried. Slides were denatured in 70% formamide with 30% 2×SSC for 160 seconds at 75°C. This was followed by quenching the slides in ice-cold 70% ethanol, then at room temperature in 80% ethanol and finally in 100% ethanol. Probe mixture was placed on the slide, covered with a coverslip and sealed. The slides were placed in a pre-heated humid chamber and incubated overnight at 37°C. After incubation, the slides with coverslip were placed in 2×SSC at room temperature for 5 minutes. After removal of the coverslip, slides were then rinsed twice in 50% formamide and 50% 2×SSC for 10 minutes at 37°C, followed by rinsing in 2×SSC with 0.1% Triton X-100 at room temperature for 1 minute. Subsequently, the slides where placed 1 hour in 4×SSC with 0,05% Triton X-100. Finally, the slides were placed in 4×SCC, 0.05% Triton X-100, 3% BSA for 25 minutes at room temperature. Slides were incubated with anti-biotin-labelled Cy3 and anti-digoxigenin Avidine Alexa Fluor 488-labelled antibodies (Invitrogen) in a dark humid chamber for 35 minutes at room temperature. After removing the coverslips, slides were washed 3 times for 3 minutes in 4×SSC with 0.05% Triton X-100, rinsed in distilled water and air dried before a droplet of Vectashield mounting medium with DAPI (4′,6′-diamidino-2-phenylindole) (Vector Laboratories) was placed on the slide and covered with a coverslip.

### Fluorescence Microscopy, Digital Image Preparation, and Analysis

Analysis of the chicken oocyte nuclei was performed using a Carl Zeiss Axioplan 2 imaging microscope (Jena, Germany) with a plan-neofluar objective 100×/1.3 oil immersion. Images were taken with a Coolsnap-pro digital camera (Photometrics, Waterloo, Canada). The acquired digital images were processed with Photoshop software (Adobe Systems).

## Results

### The Equalized ZW Is Completely Synapsed in Mid-Pachytene Oocytes

**Figure 1:**
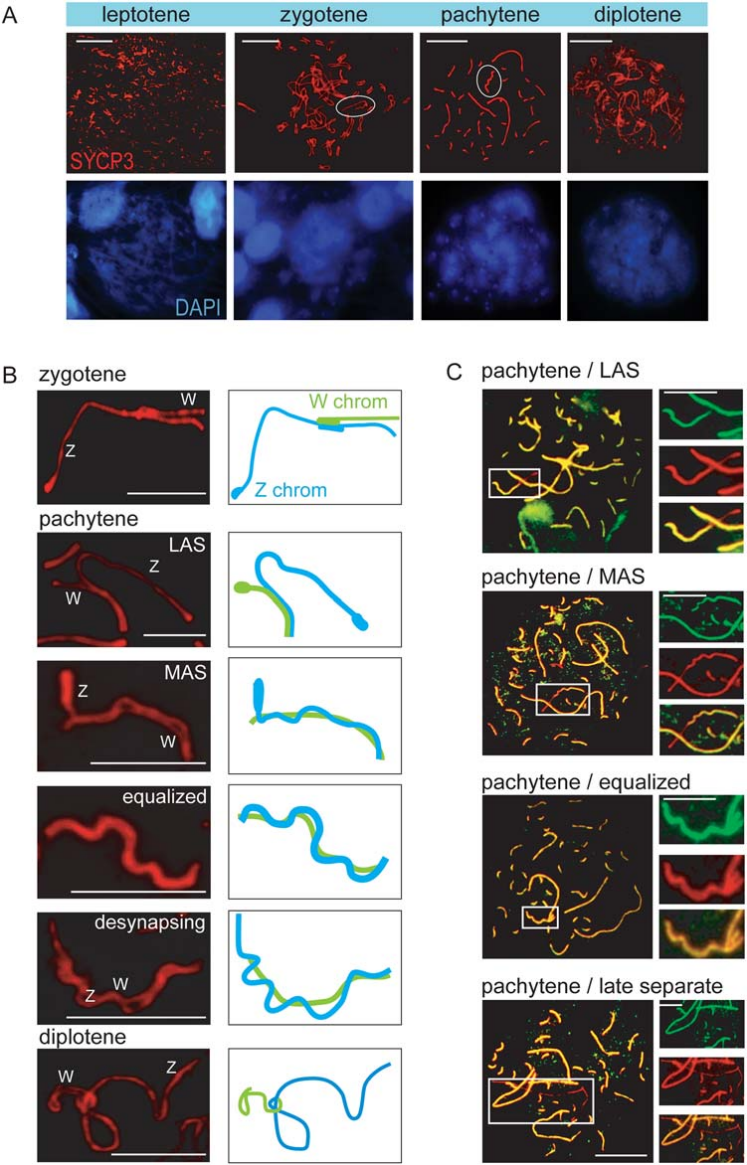
Synaptonemal complex formation and ZW pairing during meiotic prophase in chicken oocyte nuclei. A) Overview of meiotic prophase in chicken oocytes. The upper panel shows the different substages, based on the morphology of the lateral elements of the synaptonemal complexes immunostained for SYCP3 (red). The lower panel shows the corresponding DAPI stained nuclei. The ZW pair is encircled in the zygotene and pachytene oocytes. Bar represents 10 µm. B) Overview of the different synaptic configurations of the ZW pair during zygotene, pachytene, and diplotene, visualized by anti-SYPC3 (red). LAS = long asynaptic segment, MAS = medium asynaptic segment [38]; W indicates W chromosome, Z indicates Z chromosome. The panels on the right show schematic drawings of the morphological configurations of Z and W (Z chromosome in blue, W chromosome in green). Bar represents 5 µm. C) Progress of ZW synapsis during pachytene visualized by immunostaining for SYCP1 (green) and SYCP3 (red). Bar represents 10 µm. The higher magnifications show separate immunostainings for SYCP1 (green, upper), SYCP3 (red, middle) and the merge (bottom) for the ZW pair. Bar represents 5 µm.

**Figure 2:**
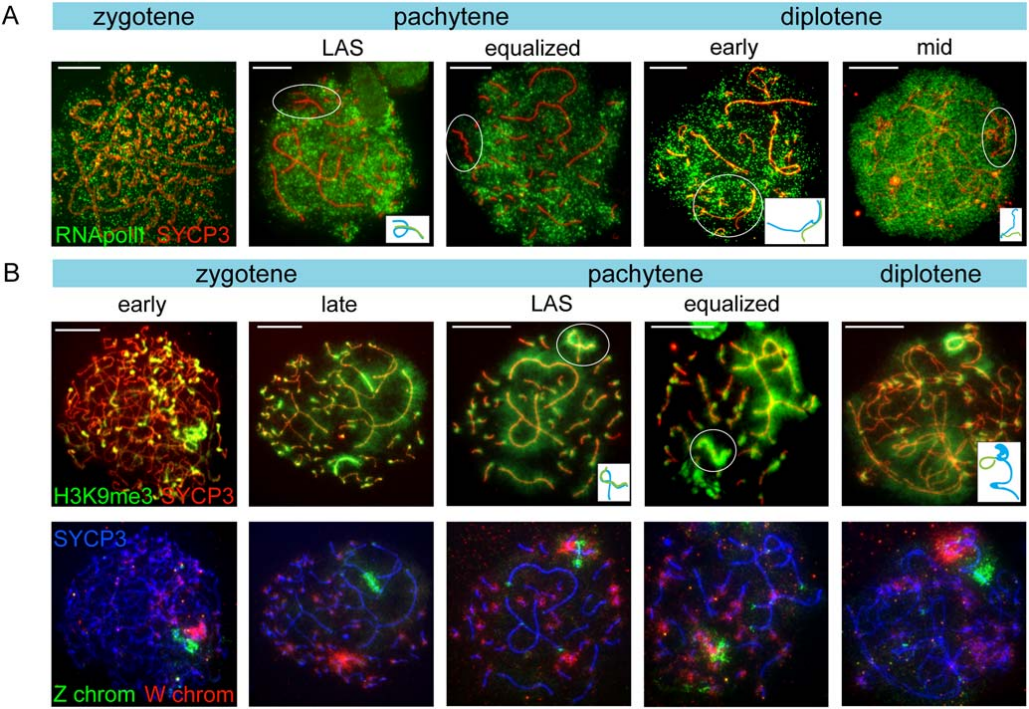
Lack of RNA polymerase II and enrichment for H3K9me3 mark the ZW pair. A) Oocyte spread nuclei immunostained for RNA polymerase II (green) and SYCP3 (red). The RNA pol II signal is evenly spread in the zygotene nucleus. In pachytene nuclei (LAS and equalized configuration of the ZW pair) and in diplotene, RNA pol II signal is reduced around the ZW pair. Bar represents 10 µm. B) Oocyte spread nuclei immunostained for H3K9me3 (green) and SYCP3 (red) (upper panel), and DNA FISH with painting probes for the heterochromatic part of the Z (green) and W (red) chromosomes (lower panel). W and the heterochromatic part of Z are enriched for H3K9me3 already in early zygotene. The highest H3K9me3 signal is seen on the fully synapsed ZW pair in pachytene. In diplotene, as Z and W have desynapsed, H3K9me3 remains highly positive on W and is lost from Z, with the exception of the constitutive heterochromatic part of the Z that is recognized by the painting probe which is still positive for H3K9me3, although with much lower intensity than W. Bar represents 10 µm.

We analysed the progression of meiotic prophase in chicken oocytes by immunostaining for SYCP3, which visualizes the lateral axial elements of the synaptonemal complex (SC). At leptotene, small SYCP3 fragments started to appear throughout the nucleus (Figure 1A). In addition to Z and W, the chicken genome is distributed over 38 autosomal chromosome pairs, including 10 pairs of microchromosomes. During zygotene, most microchromosomes are found at the periphery of one part of the nucleus, where they are aligned and have initiated pairing, whereas macrochromosomes are more confined to the center and opposite site of the nucleus, and appear entangled and disorganized (Figure 1A). At early pachytene, when all autosomes have completed synapsis, the ZW pair starts to synapse (Figure 1A,B). Around mid-pachytene, the ZW pair reaches the complete synapsed or so-called equalized stage (Figure 1B), and frequently localizes to the periphery of the nucleus (Figure 2A). These findings are consistent with the configurations of the Z and W chromosomes described by Solari [5] (Figure 1B), and we used the consecutive configurations of the ZW pair to subdivide the pachytene stage.

During early pachytene, the Z and W chromosomes appear to be separate (‘early type’). This is followed by ZW pairing and synapsing in the short pseudo-autosomal regions. Subsequently the long asynaptic segment (LAS) of Z, starts to condense and shorten (most likely by folding back on itself), becoming the medium asynaptic segment (MAS). At mid-pachytene, the Z chromosome starts to wind itself around the relatively straight W axis, resulting in a fully equalized ZW pair (Figure 1B). Next, the Z and W start to desynapse and rapidly separate again (‘late separate’). At early diplotene, Z and W display end-to-end attachment (Figure 1B). Subsequently, all bivalents desynapse, elongate and become intertwined, making it almost impossible to distinguish and follow the individual Z and W chromosomes. However, in some diplotene nuclei, the Z and W chromosomes were found to display an end-to-end pairing in a typical ζ (zeta)-like configuration (Figure 1B and 2B).

Next, we investigated if the Z and W chromosomes actually reach a state of full synapsis during the equalized stage. For this purpose, we stained for SYCP1. In contrast to SYCP3, which localizes to the chromosomal axes of meiotic chromosomes, SYCP1 is a component of the central element of the SC, which is only assembled on completely synapsed chromosomes (reviewed in [25]). During the LAS and MAS stages, SYCP1 stains only the synapsed regions of the ZW pair. As soon as the Z chromosome starts to wrap itself around the W chromosome, we observed that the SYCP1 signal followed the twists of the Z chromosome (Figure 1C). At the equalized stage, the SYCP3 and SYCP1 staining fully overlapped, except for the occasionally free tip of the Z chromosome. As pachytene progresses further, Z and W begin to desynapse, and this was accompanied by disappearance of SYCP1 from these regions (Figure 1C). At the ‘late separate’ stage, SYCP1 was no longer present on Z and W. Based on these observations, we conclude that the equalized stage indeed represents a completely synapsed configuration of Z and W.

### The Equalized ZW Chromosome Pair Is Transcriptionally Silent in Pachytene Oocytes

To analyse the transcriptional activity of the Z and W chromosomes during the different stages of meiotic prophase, we immunostained oocytes for RNA polymerase II (RNA pol II) and SYCP3. During leptotene and zygotene, we found positive staining for RNA pol II throughout the nucleus, but from early pachytene onwards, there is a depletion of RNA pol II surrounding the ZW pair (Figure 2A). The absence of RNA pol II was most prominent during the equalized stage. As pachytene progresses into diplotene, the exclusion of RNA pol II around the ZW pair persists, and a reduction of RNA pol II surrounding the SC was also observed for other macrobivalents (Figure 2A). In mid diplotene, the overall signal of RNA pol II increased, but the level in the area around ZW remained relatively low. These data indicate that the Z and W chromosomes are subjected to meiotic silencing. In late diplotene, RNA pol II staining is no longer reduced on Z and W (not shown).

To obtain further evidence for transcriptional silencing of Z and W during chicken oogenesis, we analysed the localization of the known heterochromatin marker H3K9me3 [26], in combination with a FISH specific for the W chromosome and the heterochromatic part of the Z chromosome. In oogonia, and in leptotene and zygotene oocytes, we observed several regions enriched for H3K9me3, but the region with the highest signal always colocalized with the FISH signal for W (Figure 2B). The Z painting probe colocalized with a region of Z that was also enriched for H3K9me3, but to a lesser extent compared to the enrichment of H3K9me3 on the W chromosome. During the equalized stage, the chromatin surrounding the ZW pair could easily be recognized as the region that displayed the strongest H3K9me3 staining in the nucleus (Figure 2B). As the ZW pair desynapses, H3K9me3 is lost from the Z chromosome, with the exception of the heterochromatic region that is recognised by the painting probe (Figure 2B). These findings indicate that the W chromosome is already inactive before entry into meiotic prophase, while the inactivation of the whole Z seems to be a transient process from early pachytene until diplotene. Based on our observations and earlier reports, we estimate that the duration from pachytene till early diplotene takes approximately 3–4 days [27].

### γH2AX Appears in Two Separate Waves during Meiotic Prophase

**Figure 3:**
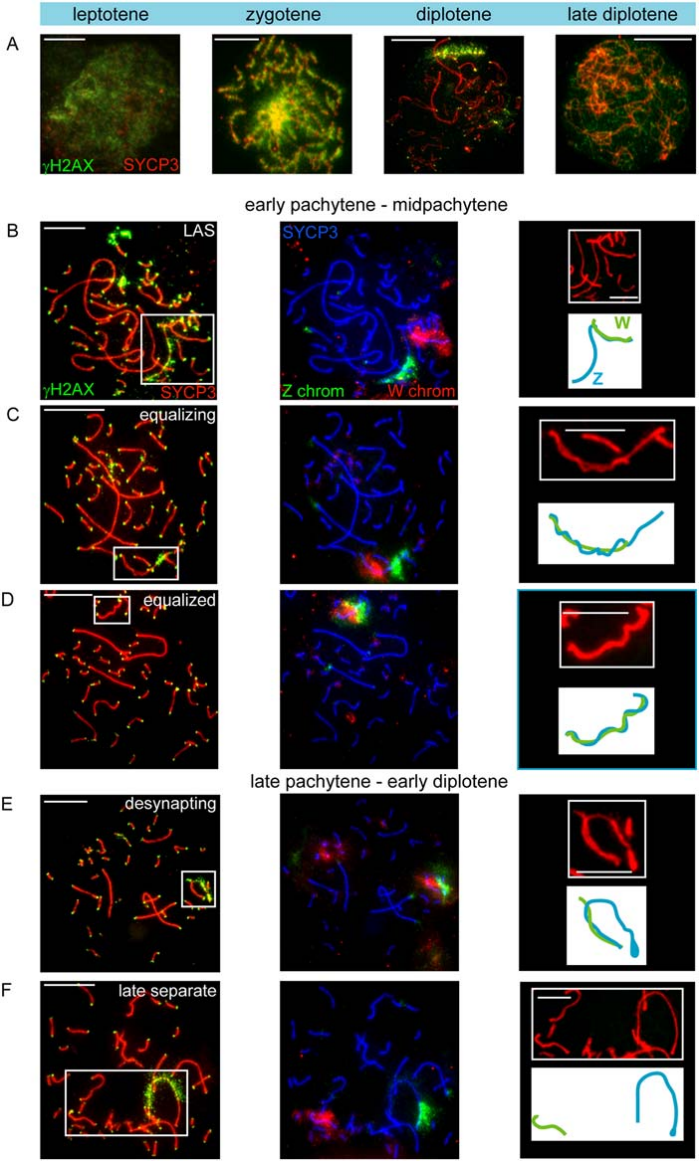
Two separate waves of γH2AX on Z during meiotic prophase in chicken oocytes. A–F) A: Oocyte spread nuclei immunostained for γH2AX (green) and SYCP3 (red) B–F: Oocyte spread nuclei immunostained for γH2AX (green) and SYCP3 (red) (left panels), DNA FISH with painting probes for the heterochromatic part of the Z (green) and W (red) chromosomes (middle panels) and schematic drawings of the synaptic configurations of Z (blue) and W (green) (right panels). At leptotene, γH2AX starts to appear, and in zygotene it is present throughout the nucleus (A). In pachytene, γH2AX marks all telomeres and is present as big foci on several chromosomes (B). γH2AX also coats the unsynapsed part of Z during early pachytene, but disappears from Z as synapsis between Z and W progresses (BC). At the equalized stage, around midpachytene, only the telomeres are γH2AX positive (D). Upon unwinding or desynapsis of the Z and W, a second wave of γH2AX starts to coat the desynapsed part of Z (E). The heterochromatic part of Z has lost γH2AX from late pachytene onwards (F). In diplotene, only the telomeres and the desynapsed Z chromosome are highly enriched for γH2AX (A). At later stages of diplotene, γH2AX disappears also from the telomeres and Z (A). Bar represents 10 µm.

Next, we analysed the behaviour of histone H2AX phosphorylated at serine 139, (γH2AX), a well-known marker of DNA double strand breaks (DSBs) [17],[28]. This is also the earliest histone modification that appears on the silenced XY body in mouse (reviewed in [29]). In chicken oocytes, γH2AX was found to be present throughout the nucleus with areas showing more intense staining in leptotene and zygotene (Figure 3A). These areas most likely correspond to sites of meiotic DSBs, similar to what has been observed for mouse oocytes and spermatocytes [17]. At the end of zygotene, remaining γH2AX foci localize to sites associated with synaptonemal complexes (SCs). Persistent γH2AX foci were observed along the unsynapsed arm of the Z chromosome (Figure 3BC), as confirmed by subsequent FISH with specific probes against the heterochromatic part of Z and the whole W chromosome. During mid pachytene, when the ZW pair is fully synapsed, γH2AX foci along the length of the SCs have disappeared, but all telomeres showed a bright focus (Figure 3D). During mid-late pachytene, when the Z and W start to desynapse, a second wave of γH2AX starts to accumulate in a distal to proximal fashion on all the desynapting regions of Z (Figure 3EF). In diplotene, γH2AX covers the whole Z chromosome, with the exception of the heterochromatic part, which looses γH2AX during the late separate stage (compare diplotene in Figure 3E with Figure 3F). In addition, all other chromosomes maintain γH2AX at the telomeres. Approximately two days after entering diplotene, as seen in part of the diplotene oocytes isolated from 7-day-old chickens, γH2AX is lost from the Z chromosome (Figure 3A).

Together with the RNA polymerase II and H3K9me3 staining patterns, these data show that the second wave of γH2AX accumulation starts after silencing of the ZW pair has been established. Moreover, the second wave of γH2AX labelling appears to be restricted to the Z chromosome. These findings contrast with observations during mouse meiosis, where γH2AX accumulation is essential for, and occurs concomitant with, silencing of the sex chromosomes [30]. To obtain more insight in the trigger for γH2AX accumulation on the Z chromosome during late pachytene in chicken oocytes, we analysed the immunolocalization of the DSB-repair protein RAD51.

### DNA Double Strand Break Repair Associated Proteins Transiently Disappear from the ZW Pair

**Figure 4:**
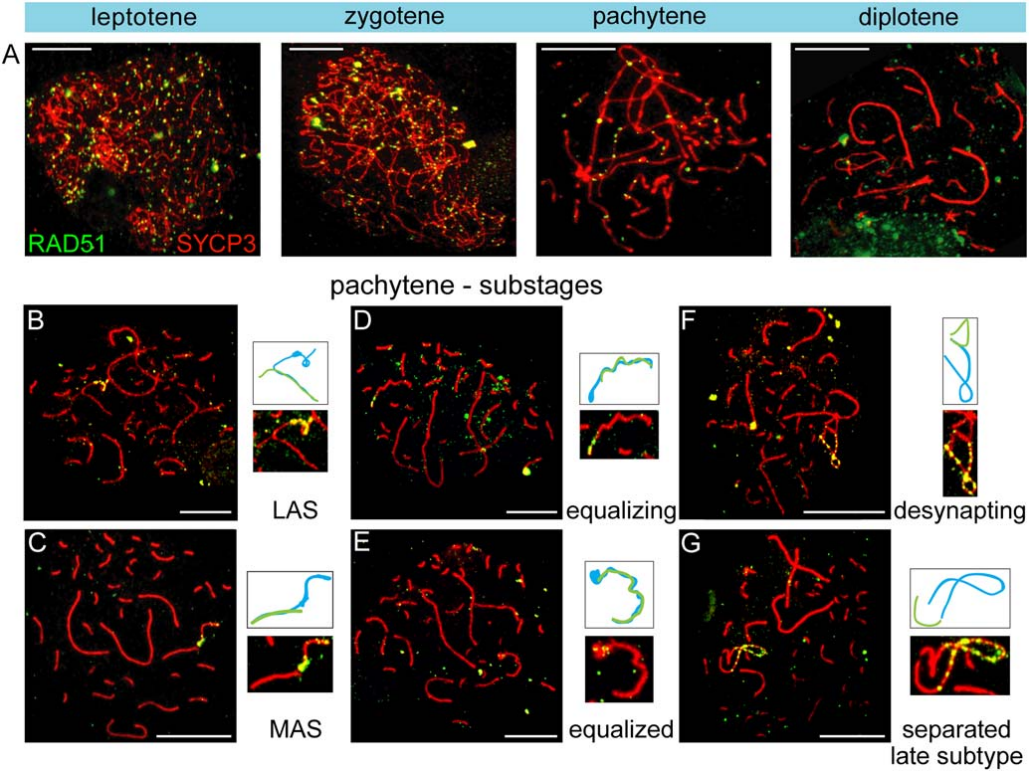
Reappearance of RAD51 foci on the desynapsing Z chromosome. A–G) Oocyte spread nuclei immunostained for RAD51 (green) and SYCP3 (red). At leptotene and zygotene, RAD51 foci are dispersed throughout the nucleus (A). At early pachytene, some RAD51 foci are present on almost all synaptonemal complexes, most prominently on the unsynapsed part of the Z chromosome (AB). With progression of pachytene, RAD51 foci disappear from the autosomes, but remain present on the unsynapsed segment of the Z chromosome (CD). As synapsis between Z and W proceeds (MAS to equalized), RAD51 foci disappear along the synapsed region of Z and W (CDE). When Z starts to unwrap itself, RAD51 foci reappear on the desynapsed part of Z (FG) and remain present until diplotene (A). Enlargements and the schematic drawings (Z in blue, W in green) of the synaptic configurations of the ZW pair are shown. Bar represents 10 µm.

We found RAD51 foci on the synapsed autosomes and the unsynapsed axis of the Z chromosome during early pachytene. However, we never observed RAD51 foci on the W chromosome during pachytene (Figure 4A–F). During progression of pachytene, RAD51 foci gradually disappeared from the autosomes. As synapsis between Z and W progresses, RAD51 foci start to disappear from the synapsed part, but remain present on the part of Z that is still unsynapsed (Figure 4B–D). During the equalized stage, only a diffuse RAD51 signal persists at the distal tip of Z (Figure 4E). When the Z and W start to desynapse, we noticed a reappearance of RAD51 foci along the desynapsing Z chromosomal axis (Figure 4F). When Z and W were almost completely separated (‘late separate’ subtype), RAD51 foci covered the complete Z chromosome (Figure 4G), whereas the W chromosome remained devoid of these foci. Upon entering diplotene, all SC-associated RAD51 foci gradually disappear (Figure 4A). The observed temporal disappearance of RAD51 foci (and γH2AX) during progression of synapsis between Z and W in pachytene could indicate that the repair proteins are transiently lost, while the breaks are not repaired. However, the lack of detectable RAD51 foci could also be due to the tight winding and twisting of Z around W, which may render the RAD51 proteins inaccessible to the antibody. Still, the absence of γH2AX from the synapsed ZW indicates that the DSBs may also be temporarily undetectable for the machinery that couples processing of these breaks to γH2AX formation. Final repair of these breaks may therefore be suppressed until Z and W desynapse.

### H2A Ubiquitylation Marks the ZW Pair from Early Pachytene until Diplotene

**Figure 5:**
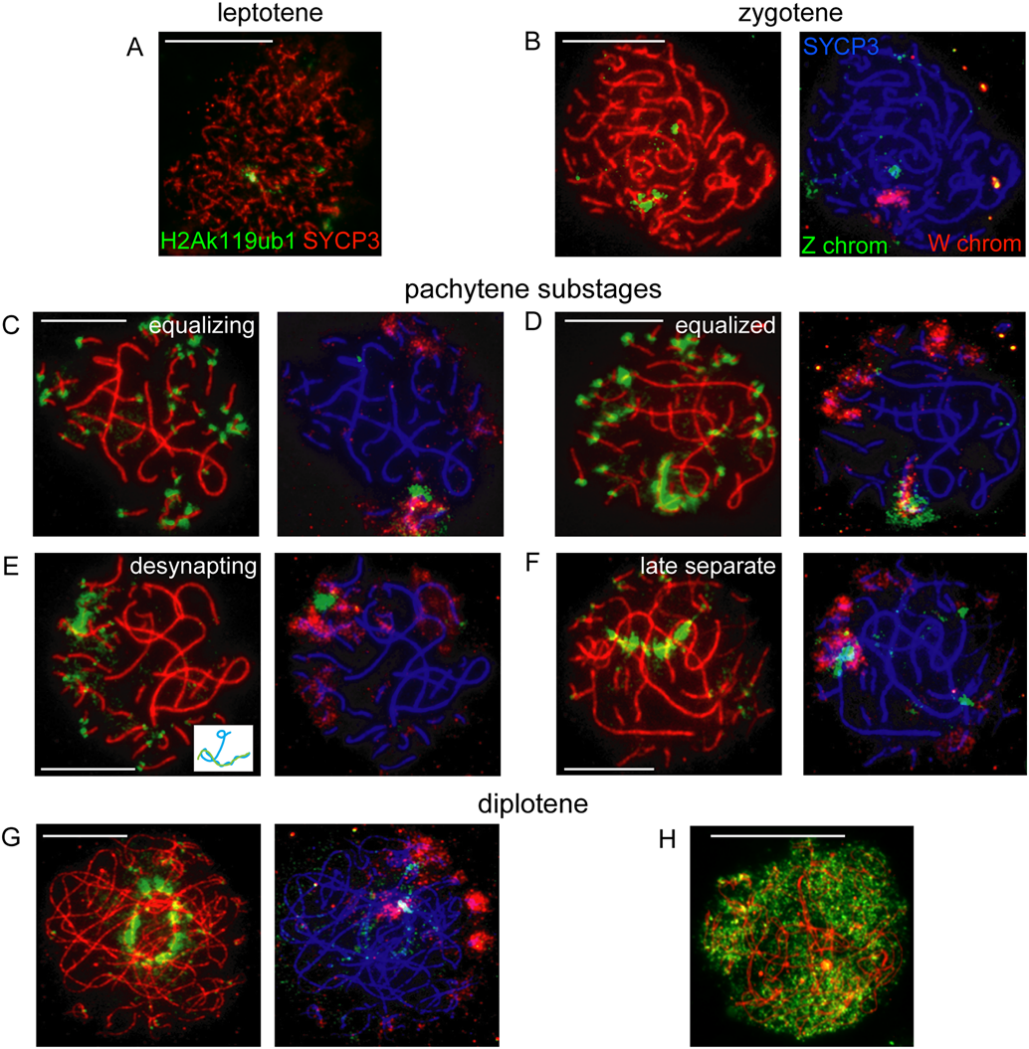
Analysis of H2Ak119ub1 on the ZW pair during meiotic prophase. A–H) A+H: Immunostaining for H2Ak119ub1 (green) and SYCP3 (red) on spread oocyte nuclei. B–G: Immunostaining for H2Ak119ub1 (green) and SYCP3 (red) on spread oocyte nuclei (left) and DNA FISH with painting probes for the heterochromatic part of the Z (green) and W (red) chromosomes (right). In leptotene, some H2Ak119ub1 areas are visible (A). H2Ak119ub1 already marks the W chromosome during early zygotene (B) and accumulates at all centromeres at late zygotene (not shown). In pachytene, H2Ak119ub1 still marks the centromeres, and H2Ak119ub1 starts to coat the Z and W chromosomes, covering both parts of the synapsed regions as well as the heterochromatic part of the unsynapsed Z (C). The ZW pair is completely covered by H2Ak119ub1 at its equalized stage (D). When the Z and W start to desynapse, H2Ak119ub1 only persists on the Z chromosome (E) (inset shows schematic drawing of Z (blue) and W (green) pair). At late pachytene, H2Ak119ub1 remains present on the Z, but is lost from its heterochromatic part (F). In diplotene oocytes from 7-day-old chickens (G), H2Ak119ub1 briefly persists on Z, but eventually disappears and is distributed throughout the nucleus (H). Bar represents 10 µm.

Next, we investigated the localization of several known other mammalian and marsupial XY body markers in chicken oocyte nuclei. First, we evaluated H2Ak119ub1, a histone modification which is generally associated with gene silencing, in combination with FISH for Z and W. It marks the inactive X chromosome in female somatic cells [13],[31],[32], and the mammalian XY body from mid-pachytene to early diplotene [33]. In chicken zygotene oocytes, this histone modification marks the W chromosome (Figure 5B) and from late zygotene onwards also accumulates on centromeric chromatin. In early pachytene, H2Ak119ub1 starts to spread from the centromeres on a few microbivalents. It also begins to accumulate on the already synapsed part of the ZW pair, and part of the distal unsynapsed arm of the Z chromosome (Figure 5C). Around mid-pachytene, when the ZW pair is fully synapsed, the H2Ak119ub1 signal increases and intensifies specifically on the ZW pair (Figure 5D). When the Z and W start to desynapse, H2Ak119ub1 remains present on the desynapting Z chromosomal axis, but is eventually lost from the W (Figure 5E). At the late separate stage and during early diplotene, H2Ak119ub1 covers the Z chromosome (Figure 5FG), with exception of the heterochromatic part recognized by the FISH probe. A few days after entering diplotene, H2AK119ub1 is lost from the Z chromosome, and appears in an evenly distributed manner throughout the whole nucleus (Figure 5H).

### Trimethylation of Lysine 27 of Histone H3 Is a Prominent Marker of the W Chromosome during Meiotic Prophase

**Figure 6:**
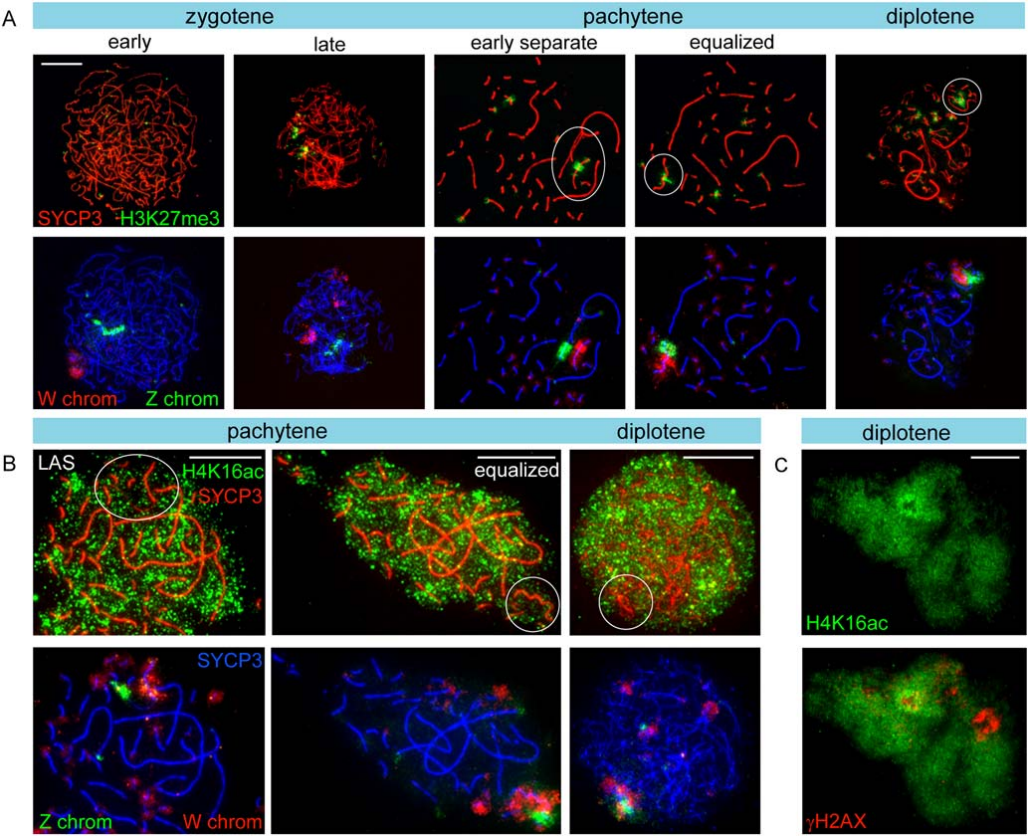
Analysis of histone modifications on Z and / or W during meiotic prophase. A) Immunostaining of oocyte spread nuclei preparations for H3K27me3 (green) and SYCP3 (red) (upper panel) and DNA FISH with painting probes for the heterochromatic part of the Z (green) and W (red) chromosomes (lower panel). With progression of zygotene, part of the W chromosome becomes positive for H3K27me3. During pachytene, H3K27me3 is present on several microbivalents and coats a specific part of the W chromosome. This pattern persists up into diplotene. Bar represents 10 µm. B) Oocyte spread nuclei preparations immunostained for H4K16ac (green) and SYCP3 (red). From around midpachytene onwards, H4K16ac is reduced around the ZW pair. In pachytene, also other chromosomes appear to have reduced levels of H4K16ac. Bar represents 10 µm. C) Oocyte spread nuclei preparations immunostained for H4K16ac (green) and γH2AX (red). In diplotene, γH2AX and H4K16ac signals are mostly mutually exclusive. Bar represents 10 µm.

H3K27me3, an early marker of X chromosome inactivation in the female mouse embryo [34], is reduced on the XY body in mammals [35] and marsupials [36]. In chicken leptotene oocyte nuclei, this modification is virtually absent, whereupon the signal in zygotene nuclei increases on W and some microbivalents (Figure 6A). During pachytene, H3K27me3 is enriched on three microbivalents, but this modification most prominently marks a subregion of the W chromosome (Figure 6A). This enrichment of H3K27me3 on the W chromosome was found to remain prominent only on the W chromosome, even in late diplotene (Figure 6A).

### Acetylation of H4K16 on the ZW Pair Is Reduced from Mid Pachytene until Diplotene

Acetylation of H4K16 is associated with active transcription, and in nuclei of female chicken somatic cells, a subregion of the Z chromosome is specifically enriched for this histone modification [37]. We performed double-immunostainings of oocytes for H4K16ac and SYCP3, followed by a FISH for Z and W. During zygotene, H4K16ac stained the nucleus more prominent then during leptotene and pachytene, which could indicate a transient global upregulation of transcription (not shown). Similar to what was observed for RNA polymerase II, reduced H4K16ac staining is observed on the completely synapsed ZW pair (Figure 6B). As pachytene progresses, H4K16ac is also reduced around long autosomal SCs. The low level of H4K16ac on ZW appears to persist until mid diplotene. We also performed double-stainings for γH2AX and H4K16ac and observed that when γH2AX accumulates on the desynapting Z, H4K16ac is reduced, and this persists up to diplotene (Figure 6C). Concomitantly, H4K16ac signal increases on the rest of the genome. Together, these observations show that the Z and W chromosome lose H4K16ac around the midpachytene stage, indicating transcriptional silencing, in accordance with our other observations.

### mRNAs of Z and W Genes Show Transient Downregulation in Oocytes during Early Postnatal Ovary Development

**Figure 7:**
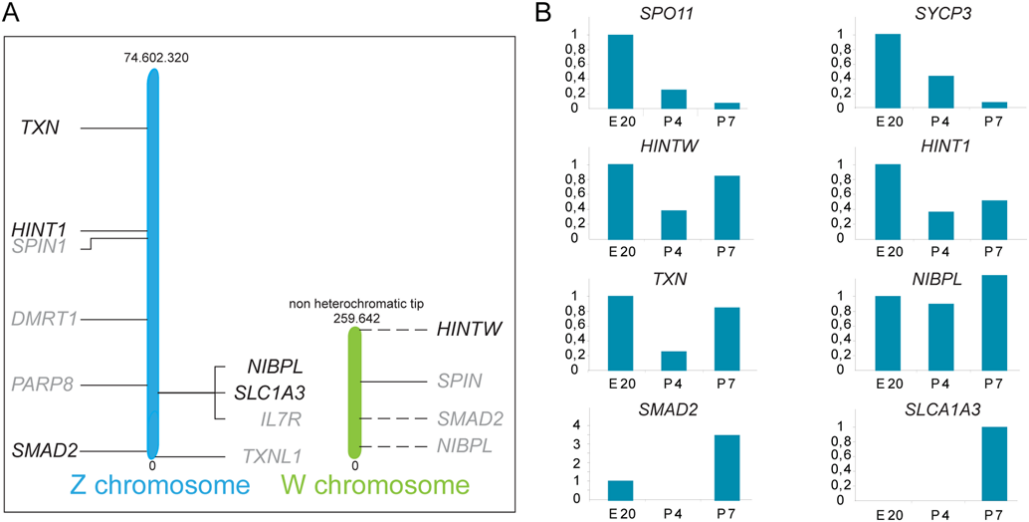
Gene expression profile in oocytes in different stages of meiotic prophase. A) Schematic drawing of the location of the analyzed genes on the Z and W chromosomes. The genes in gray did not show expression in oocytes at any of the analysed timepoints. The intermittent lines from the W chromosome indicate that the exact location of the genes is unknown [42]. B) Gene expression graphs as analyzed by real time RT PCR for two autosomal meiosis-specific genes; *SPO11* and *SYCP3*, for 1 W chromosome gene (*HINTW*) and 5 Z chromosomal genes (*HINT1*, *TNX*, *NIBPL*, *SMAD2*, and *SLCA1A3*). Data were collected at 3 different time-points: embryonic day 20 (E20), 4 (P4) and 7 days post-hatching (P7), and expression in oocytes was estimated as described in Materials and Methods. Finally, expression at E20 was set at 1, except for *SLCA1A3*, which was expressed in oocytes only on day 7 post hatching.

If the Z and W chromosome are silenced during pachytene and early diplotene, mRNAs for Z and W-encoded genes should be decreased in these cells, compared to earlier and/or later stages of oocyte development. To analyse this, we performed real-time RT-PCR experiments using total RNA isolated from purified oocyte fractions and total ovaries isolated on 3 different time points (embryonic day 20, post hatching day 4 and day 7). Real time RT-PCR was performed for 10 Z-encoded genes, 4 W-encoded genes (Figure 7A) and 2 autosomal meiosis-specific genes, namely the synaptonemal complex component *SYCP3* and meiotic-DNA double strand break-inducing enzyme *SPO11*. The expression profiles of *SYCP3* and *SPO11* followed the expected pattern (Figure 7B, Figure S1). The Z genes, *HINT1*, *TXN*, *NIPBL* and *SMAD2* all show a relative decrease in expression in oocytes of post hatching day 4, followed by an increase in expression at day 7 (Figure 7B); Expression of the Z gene *SLCA1A3* is measured in oocytes for the first time at day 7. The W gene *HINTW* also shows a decrease on post hatching day 4, and subsequently increased expression at day 7. The other analysed Z- en W-encoded genes showed no expression in oocytes at any timepoint, indicating that they are inactive during meiotic prophase. Based upon earlier descriptions of ovary development [5],[10],[38] and our own observations, most oocytes are still in zygotene during embryonic day 20, whereas the vast majority of the oocytes is in late pachytene on post hatching day 4, and in late diplotene on day 7. The observed decrease in mRNA levels of Z- and W-encoded genes supports our immunocytochemical findings and confirms that Z and W are transiently silenced during oocyte development.

### The ZZ Chromosome Pair Behaves Similar to the Autosomal Chromosome Pairs during Male Meiotic Prophase

To establish that the ZW pair in oocytes behaves different from the ZZ pair in spermatocytes, we also performed immunocytochemical analyses on chicken spermatocytes isolated from adult testes. Similar to what we observed in oocytes, , γH2AX was present throughout the nucleus with areas showing more intense staining in leptotene and zygotene spermatocytes (Figure S2A). At the end of zygotene, remaining γH2AX foci localize to sites associated with synaptonemal complexes, also resembling the pattern observed in chicken oocytes. However, during pachytene, all chromosomes were fully synapsed and γH2AX was present only on telomeres, and this pattern persisted up to late diplotene (Figure S2A).

Next, we analyzed the presence of H3K9me3 in combination with a FISH specific for the heterochromatic regions of the Z chromosomes (Figure S2B). Several regions in leptotene and zygotene spermatocytes were enriched for H3K9me3, but they never colocalized with the FISH signal(s) of Z (Figure S2B). In pachytene, the heterochromatic region of Z showed some enrichment for H3K9me3, and this signal decreased again during diplotene (Figure S2B).

H3K27me3 was present on a few microchromosome throughout meiotic prophase, but not on Z (not shown).

## Discussion

### Meiotic Inactivation of the Synapsed ZW Pair in Chicken Oocytes

Meiotic sex chromosome inactivation (MSCI) in male mammals is thought to be triggered by the presence of unsynapsed axes of the X and Y chromosome (reviewed in [29]). Recently, it was discovered that in marsupial spermatocytes the unsynapsed X and Y chromosomes are also inactivated in a manner similar to what has been observed in mouse [36],[39]. Herein, we show inactivation of sex chromosomes during meiosis in the female *Gallus gallus domesticus*, a species with female heterogamety and a ZW sex chromosome system that evolved independent of XY. Female oocytes undergo a much longer developmental process between the initiation of meiotic prophase and ovulation, compared to the time course that is involved during development of spermatocytes to mature sperm. Therefore, it was suggested that meiotic inactivation of Z (and W) would not occur because it would be incompatible with the lengthy oocyte developmental process [12]. Herein, we have shown to the contrary that MSCI does occur, but is transient in chicken oocytes; in diplotene, the Z chromosome loses its specific “silencing” histone modifications (γH2AX and H2Ak119ub1). In addition, the mRNA of several Z-encoded genes is higher in oocytes isolated at posthatching day 7 compared to day 4. Reactivation of Z may allow Z-encoded genes to assist in further oocyte development. *HINTW* is a W chromosomal multicopy gene [40],[41] that also shows increased expression in day 7 oocytes. It localizes to the non-heterochromatic tip of W [42]. Based on its female specificity and expression in differentiating ovaries of early embryos, *HINTW* has been implicated in female sex differentiation, but its exact function is unclear [42]. The W chromosome is gene poor, and to date, only a few genes have been described to be W-specific (ICBN Mapviewer, [41],[43]). In addition, the actual size of the pseudo-autosomal region between Z and W has not been established. Based on the persistent presence of H3K9me3 on W in diplotene oocytes, it could be suggested that the W remains inactive throughout oocyte development, perhaps with the exception of the non-heterochromatic tip that contains the multicopy gene *HINTW*. This nicely parallels the recent findings by Mueller et al [44], who describe that X- linked multicopy genes that are subjected to MSCI are specifically re-expressed in postmeiotic spermatids in mouse, whereas the vast majority of single-copy genes remain inactive.

In early mouse pachytene spermatocytes, the X and Y chromosome show more extensive synapsis compared to the later pachytene stages, when desynapsis progresses until the X and Y show only an end to end association in some diplotene nuclei [45]. This resembles the dynamics of ZW association during chicken oogenesis, with exception of the fact that complete synapsis is never achieved in mouse, and always in chicken. Our data now show that despite the complete (heterologous) synapsis, sex chromosome inactivation is not prevented, and repair of meiotic DSBs is delayed (see below).

### No Compensation for Z Inactivation by Retrogene Expression from Autosomal Sites

During mammalian MSCI, silencing of some essential X chromosomal genes is compensated by expression from retroposed copies on autosomal chromosomes. The expression of these copies is male-specific and initiates concomitant with MSCI [46]. However, in the chicken genome, very few functional retroposed genes appear to be present [47]. For the 15 identified functional retroposed genes in chicken, no bias for genes from specific chromosomes was detected. Due to the transient nature of the ZW inactivation, Z-encoded mRNAs and proteins may be in large enough supply to allow maintenance of function of essential Z-linked genes during this short period. Genomic analyses and analyses of EST databases have revealed that ovary-specific genes are underrepresented on the chicken Z chromosome. In addition, microarray analyses of gene expression in different chicken tissues have shown that the average expression of Z-linked genes versus autosomal genes is lowest in the embryonic ovary [48]. This phenomenon could have different causes. In principle, so-called sexually antagonistic genes (genes beneficial to one sex, detrimental to the other), are expected to accumulate on the sex chromosomes. In species with male heterogamety, recessive male beneficial genes would be expected to accumulate on the X. In accordance with this notion, the mouse and human X chromosome are enriched for spermatogenesis-genes expressed prior to meiotic prophase. Due to MSCI and PMSC (post meiotic sex chromatin), the X is depleted for spermatogenesis-genes expressed during later stages, with the exception of some single-copy and multicopy genes, that show postmeiotic reactivation [44],[49],[50]. Since retroposition of Z genes to autosomes does not seem to occur in chicken [47], it might be suggested that the evolutionary force to drive oocyte-specific genes off the Z during evolution is relatively weak, perhaps due to the transient nature of MSCI in chicken. Still, the relative lack of ovary-specific genes, and the generally low level of Z-encoded gene expression in embryonic ovary may indicate that MSCI in chicken reduces the likelihood of oocyte-specific genes that function during meiotic prophase to evolve on the Z. However, the properties of the chicken Z chromosome can also be explained by a dominant model of sexual antagonistic genes, whereby dominant genes encoding proteins that are beneficial to males would be downregulated in females to minimize antagonism [51]. More detailed analyses of ovary-specific genes is required, including separate analyses of genes expressed in somatic and germ line cells of the ovary, to determine whether MSCI in chicken affects gene content on Z.

### Z Inactivation Precedes the Second Wave of γH2AX Formation

**Figure 8:**
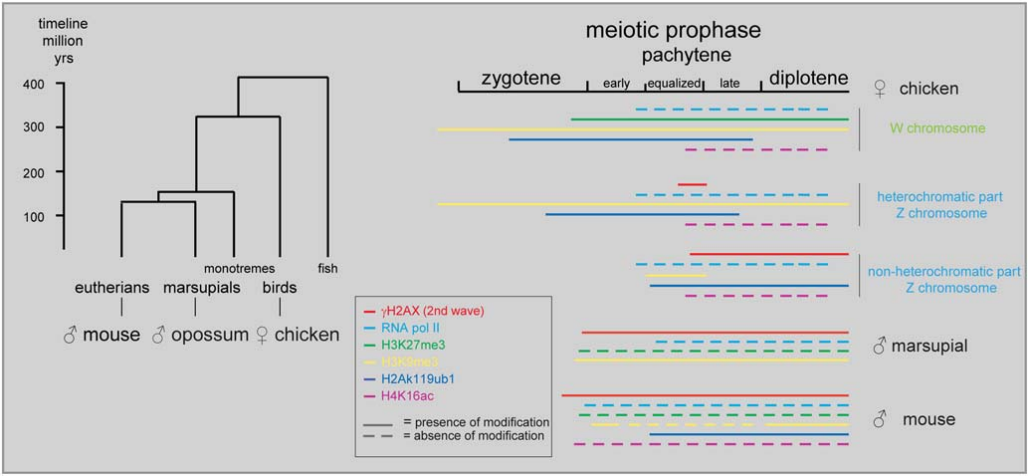
Phylogenetic tree, and overview of marsupial and mammalian XY body and chicken ZW pair markers. Timescale for the avian (chicken) and mammalian (mouse, marsupials) vertebrates [59]–[63]. Overview of the localization of different markers on XY (mouse [29],[35],[64] and marsupial [36]) and ZW (chicken) during meiotic prophase.

The inactivation of Z and W during chicken oogenesis shows marked differences and similarities to MSCI in marsupial and mouse (summarized in Figure 8). The timing of Z inactivation (early pachytene) is similar to what has been observed in the other vertebrates. However, the W chromosome appears to enter the zygotene stage already in a (partially) inactivated configuration. γH2AX appears as foci on the Z chromosome during zygotene, and these foci appear to persist longer on the Z compared to the autosomes, similar to what has been observed on the X chromosome during zygotene in mouse [17],[52]. However, during the stage of complete synapsis, γH2AX is absent from the ZW pair. Then, a second wave of γH2AX formation appears around late pachytene on the desynapsed Z, and only after silencing has been established. This is in marked contrast with the second wave of γH2AX formation in mouse, which occurs on both X and Y, and immediately as spermatocyes enter pachytene. The appearance of γH2AX on the desynapting Z chromosome is accompanied by a reappearance of RAD51. At this stage autosomal axes also begin to desynapse, but do not show a reappearance of RAD51 foci, and do not accumulate γH2AX. Thus, repair of meiotic DSBs on the Z chromosome may be inhibited to avoid recombination with the synapsed W chromosome, and postponed until desynapsis. This provides a clear link between the second wave of γH2AX formation and DSB-repair rather than with an unsynapsed axis per se. At this late pachytene/early diplotene stage, H2Ak119ub1 formation is also specifically enhanced on the Z chromosome. This modification is known to be associated with inactive chromatin, but has also been implicated in DSB-repair [53]. Perhaps silencing is induced at sites containing persistent DSBs to prevent aberrant (truncated) transcription through the damaged region in somatic as well as germ-line cells. In somatic cells, DSB repair can also be associated with the recruitment of silencing factors [54]. We reported a link between the presence of persistent DSBs and the frequency of meiotic silencing of unsynaped chromatin (MSUC) in mouse [22].

### Inactivation of Z during Pachytene Is Most Likely Accomplished by Spreading of Heterochromatin from W

**Figure 9:**
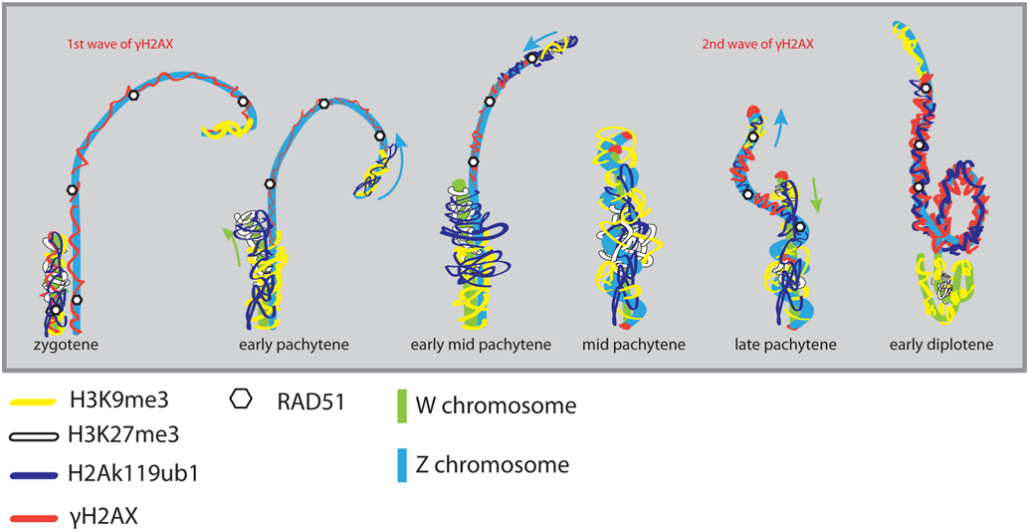
Model for transient sex chromosome inactivation in the chicken oocyte. In zygotene, parts of the W chromosome are already inactive and marked by increased H3K9me3 and H2Ak119ub1. In addition, part of the W chromosome acquires H3K27me3. The Z chromosome is enriched for H3K9me3 and H2Ak119ub1 only in the region that is known to be heterochromatic. During this phase, RAD51 foci are present and the first wave of γH2AX accumulation has occurred clearly on Z and most likely also to a lesser extent on W. Upon entrance of pachytene, Z starts to condense and folds back on itself, while the W chromosome lengthens slightly, and RNA pol II and the first wave of γH2AX gradually disappear. With progression of pachytene, the ZW pair reaches full synapsis, H3K9me3 and H2AK119ub1 most likely spread *in trans* from W on Z while H4K16ac is lost. Also, silencing may spread on Z *in cis*, triggered by the presence of persistent meiotic DSBs. As the ZW starts to desynapse, RAD51 reappears on the desynapting Z, and a second wave of γH2AX on the whole Z chromosome follows. H2AK119ub1 also spreads on the desynapting Z. During this period, meiotic DNA double-strand breaks on Z are most likely repaired, and transcriptional silencing of Z is maintained by γH2AX and H2AK119ub1. Shortly after desynapting, both γH2AX and H2AK119ub1 are lost from the heterochromatic part of Z, while H3K9me3 is lost from Z, except from its heterochromatic region. In diplotene, both sex chromosomes lengthen, and γH2AX and H2AK119ub1 are gradually lost, allowing re-activation of the Z chromosome, while W remains positive for H3K27me3 and H3K9me3, most likely indicating a maintenance of its inactive state.

With the identification of meiotic sex chromosome inactivation in a species that shows female heterogamety as well as complete nonhomologous synapsis during pachytene, we provide indications for the presence of an evolutionary force that drives meiotic sex chromosome inactivation independent of the final achievement of synapsis. The absence of homologous chromatin (as a template for the repair of DNA double-strand breaks) could be instrumental in initiating this silencing, since synapsis is only achieved after silencing has been established. Based on the observations described herein, we propose the following model for the inactivation of the sex chromosome in the heterogametic female oocyte during meiotic prophase (Figure 9): The W chromosome enters meiosis in an inactive configuration, which includes hypermethylation of H3K9 and ubiquitylation of H2AK119. H3K27me3 is also present on the W chromosome from zygotene onwards. H3K27me3 may recruit the polycomb protein complex PRC1, which could then help to enhance ubiquitylation of H2AK119, as has been observed during X inactivation in somatic cells of female mammals [32]. In pachytene, H3K27me3 remains enriched on a subregion of the synapsed ZW pair. Concomitantly, the synapsis with Z allows spreading *in trans* of heterochromatic marks such as H2Ak119ub1 and H3K9me3 from the inactive W chromosome on the Z chromosome. Also, additional spreading *in cis* of H3K9me3 and H2Ak119ub1 from the heterochromatic region of Z may contribute to inactivation of Z, triggered by the transient persistence of the meiotic DSB-associated γH2AX-signal.

The accumulated silencing histone modifications result in inhibition of Z and W gene transcription, as visualized by reduced RNA polymerase II staining around ZW, and reduced mRNA expression of selected Z and W genes. During the compact arrangement of the Z-chromosomal axis around the W axis, DSB-repair is inhibited, and γH2AX and possibly also RAD51 are lost from the DSB-repair sites. Subsequent desynapsis is accompanied by reappearance of RAD51, a second wave of γH2AX formation and enhanced H2Ak119ub1 formation on Z. The latter modification may maintain silencing (despite the absence of H3K9me3 on the desynapsed Z) until the breaks are repaired. The W chromosome remains inactive due to the high levels of H3K9me3 and H3K27me3.

Transcriptional inactivation of the ZW pair was first observed in oocytes when Z and W show the LAS to MAS configuration, at day 1 after hatching. Disappearance of γH2AX and H2Ak119ub1 from Z in diplotene was observed in oocytes isolated at the 7^th^ day after hatching. This indicates that the period of Z inactivation lasts approximately 5.5–6 days.

### Meiotic Sex Chromosome Inactivation and Dosage Compensation

A wide variety of mechanisms exist that compensate for unequal gene dosage in species with chromosomal sex determination. Female marsupials show inactivation of the paternal X chromosome in somatic cells, to equalize the expression level of X-encoded genes with that of males. The recent discovery of MSCI and maintenance of X inactivation in postmeiotic cells of male marsupials supports the hypothesis that inheritance of a “pre-inactivated” X chromosome could contribute to the establishment of paternal X-inactivation in female embryos [36]. Our findings on transient ZW inactivation argue against the existence of such a mechanism in birds. This is in accordance with data from the literature that show that male birds do not show inactivation of one of the two Z chromosomes [48],[55],[56]. In fact, dosage compensation in birds appears to be far less complete than in mammals, and it is not yet known whether dosage compensation, if it occurs, is achieved via upregulation of Z-genes in females, or downregulation in males. It cannot be excluded however, that the transient inactivation of Z leads to epigenetic modifications that persist and may influence gene expression in male (ZZ) offspring.

### Meiotic Silencing in Evolution

During male meiosis in mice, a general mechanism named meiotic silencing of unsynapsed chromatin (MSUC) silences all unsynapsed chromosomes [13],[15]. This mechanism could be evolutionary related to meiotic silencing by unpaired DNA (MSUD) which operates in *Neurospora crassa* [57]. However, MSUD is a posttranscriptional silencing mechanism that acts at the single gene level. It is not clear whether components of MSUD are conserved and used in MSUC, which acts at a much larger scale and is far less efficient. Meiotic silencing of sex chromosomes (MSCI) in mammals is most likely a specialized form of MSUC. The driving force behind MSUD and MSUC may be that it is beneficial for the species to silence foreign DNA. Although sex chromosomes are no foreign DNA, recognition as such may also be beneficial, because it will help to suppress recombination between the heterologous regions of the sex chromosomes. This suppression of sex chromosome recombination could also be a strong driving force to silence single or heterologous sex chromosomes. Spreading of heterochromatin from W on Z in female chicken oocytes to achieve meiotic sex chromosome inactivation may be mechanism that evolved independent from MSCI in mammals. In XO male grasshoppers, the single X chromosome also enters spermatogenesis in an already inactive configuration [58]. In chicken, the heterologous synapsis between Z and W may be required to escape from a synapsis checkpoint, and not to avoid meiotic silencing.

## Supporting Information

Figure S1 Gene expression profile in ovaries in different stages of meiotic prophase. Gene expression graphs with SEM as analyzed by real time RT PCR using total ovary RNA for two autosomal meiosis specific genes; *SPO11* and *SYCP3*, for 1 W chromosome gene (*HINTW*) and 5 Z chromosomal genes (*HINT1, TNX, NIBPL, SMAD2*, and *SLCA1A3*). Data were normalized to actin at 3 different time-points: embryonic day 20 (E20), 4 (P4) and 7 days post-hatching (P7). Expression at E20 was set at 1.
(0.22 MB TIF)

Figure S2 Analysis of histone modifications during meiotic prophase of male chicken spermatocytes. A. Spermatocyte spread nuclei immunostained for γH2AX (green) and SYCP3 (red). At leptotene γH2AX starts to appear and in zygotene it is present throughout the nucleus. In pachytene, γH2AX marks all telomeres, then it gradually disappears from telomeres in diplotene. Bar represents 10 micrometer. B. Spermatocyte spread nuclei immunostained for H3K9me3 (green) and SYCP3 (red) (upper panel) and DNA FISH with painting probes for the heterochromatic part of the Z (light blue) and SYCP3 (red) (lower panel). In leptotene and zygotene, H3K9m3 is present throughout the nucleus with several regions of higher signal intensity, and the Z chromosomal regions show the same H3K9me3 signal as the majority of the nucleus. In mid pachytene, some microchromosomes and the heterochromatic part of Z have a slightly higher signal. In diplotene (2 nuclei are shown), H3K9me3 signal is found in a patchy pattern on some macrochromosomes and minichromosomes, and it is lost from the heterochromatic part of Z.
(5.84 MB TIF)
