# Supplementary figures and images for: Female Meiotic Sex Chromosome Inactivation in Chicken
Source: PLoS Genet. 2009 May 22;5(5):e1000466. doi: 10.1371/journal.pgen.1000466 (PMC2678266; doi:10.1371/journal.pgen.1000466)

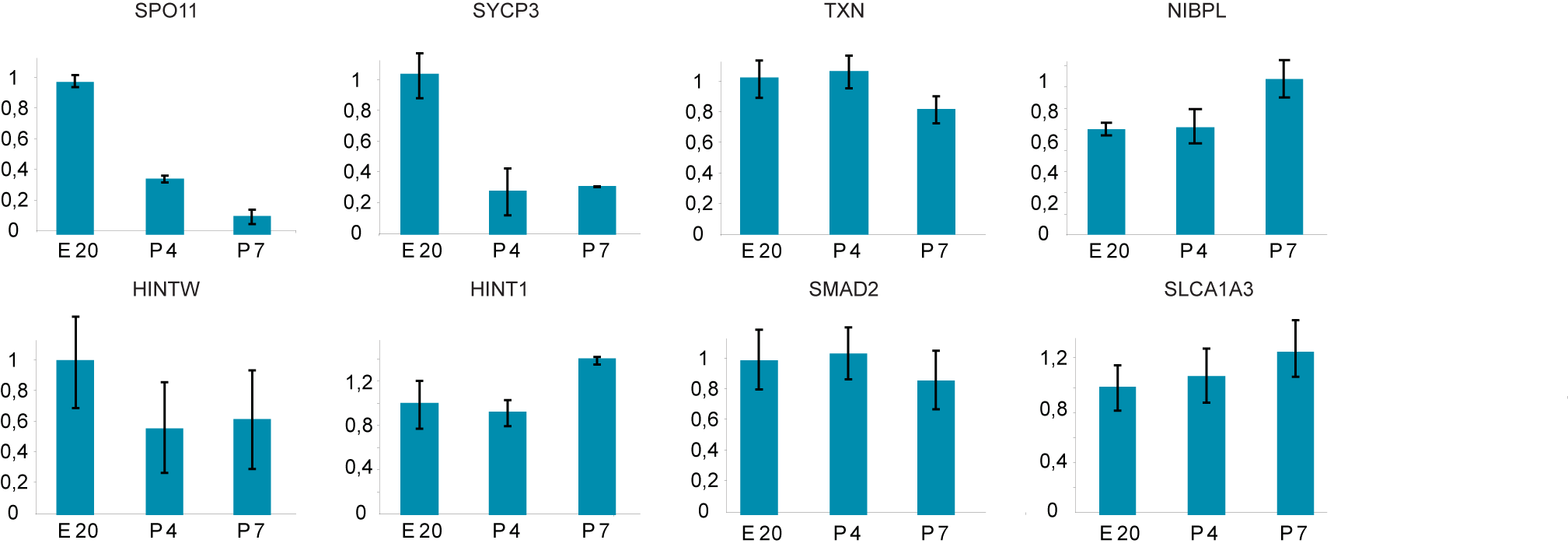

Supplement: Figure S1 — Gene expression profile in ovaries in different stages of meiotic prophase. Gene expression graphs with SEM as analyzed by real time RT PCR using total ovary RNA for two autosomal meiosis specific genes; SPO11 and SYCP3, for 1 W chromosome gene (HINTW) and 5 Z chromosomal genes (HINT1, TNX, NIBPL, SMAD2, and SLCA1A3). Data were normalized to actin at 3 different time-points: embryonic day 20 (E20), 4 (P4) and 7 days post-hatching (P7). Expression at E20 was set at 1. (0.22 MB TIF) [file pgen.1000466.s001.tif]

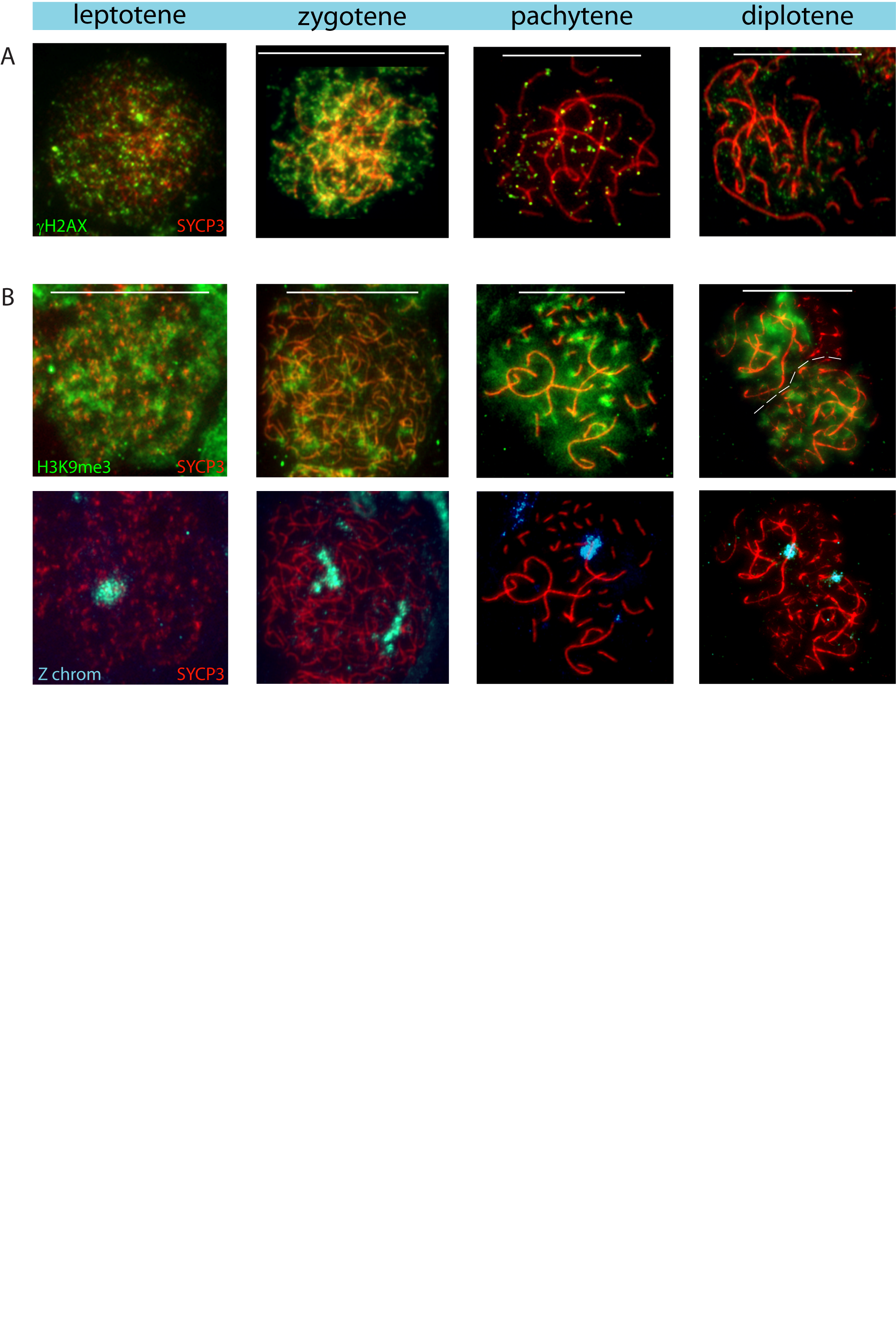

Supplement: Figure S2 — Analysis of histone modifications during meiotic prophase of male chicken spermatocytes. A. Spermatocyte spread nuclei immunostained for γH2AX (green) and SYCP3 (red). At leptotene γH2AX starts to appear and in zygotene it is present throughout the nucleus. In pachytene, γH2AX marks all telomeres, then it gradually disappears from telomeres in diplotene. Bar represents 10 micrometer. B. Spermatocyte spread nuclei immunostained for H3K9me3 (green) and SYCP3 (red) (upper panel) and DNA FISH with painting probes for the heterochromatic part of the Z (light blue) and SYCP3 (red) (lower panel). In leptotene and zygotene, H3K9m3 is present throughout the nucleus with several regions of higher signal intensity, and the Z chromosomal regions show the same H3K9me3 signal as the majority of the nucleus. In mid pachytene, some microchromosomes and the heterochromatic part of Z have a slightly higher signal. In diplotene (2 nuclei are shown), H3K9me3 signal is found in a patchy pattern on some macrochromosomes and minichromosomes, and it is lost from the heterochromatic part of Z. (5.84 MB TIF) [file pgen.1000466.s002.tif]
